# Body mass index has a non-linear association with three-month outcomes in men with acute ischemic stroke: An analysis based on data from a prospective cohort study

**DOI:** 10.3389/fendo.2022.1041379

**Published:** 2022-12-12

**Authors:** Yibing Zan, Wei Xiong, Xiaohua Zhang, Yong Han, Changchun Cao, Haofei Hu, Yulong Wang, Haining Ou

**Affiliations:** ^1^ Department of Rehabilitation, Shenzhen Dapeng New District Nan’ao People’s Hospital, Shenzhen, Guangdong, China; ^2^ Guangzhou Medical University, Guangzhou, Guangdong, China; ^3^ Department of Rehabilitation, Shenzhen Second People’s Hospital, The First Affiliated Hospital of Shenzhen University, Shenzhen, Guangdong, China; ^4^ Department of Emergency, Shenzhen Second People’s Hospital, Shenzhen, Guangdong, China; ^5^ Department of Nephrology, Shenzhen Second People’s Hospital, Shenzhen, Guangdong, China

**Keywords:** prognosis, acute ischemic stroke, non-linear association, generalized additive model, smooth curve fitting

## Abstract

**Objective:**

Despite reports of a connection between body mass index (BMI) and stroke outcome, the findings remain debatable. In this investigation, we sought to determine whether BMI was associated with the probability of 3-month unfavorable outcomes in patients with acute ischemic stroke (AIS).

**Methods:**

This is a second analysis based on a cohort study. 1,897 people with AIS who were treated at a hospital in South Korea from January 2010 to December 2016 were included in the study. The linear relationship between BMI and unfavorable outcomes for AIS patients was evaluated using a binary logistic regression model. The generalized additive model (GAM) and smoothed curve fitting (penalized spline approach) were employed to see if there was a non-linear association between BMI and unfavorable outcomes in patients with AIS.

**Results:**

The binary logistic regression model did not detect any statistically significant correlation between BMI and unfavorable outcomes in AIS patients after controlling for variables. The association between them, however, was non-linear, with the BMI inflection point occurring at 23.07 kg/m^2^. Each 1 kg/m^2^ rise in BMI on the left side of the inflection point was linked to a 12% lower risk of unfavorable outcomes (OR= 0.88, 95% CI: 0.82 to 0.96, p = 0.003). We stratified the AIS patients by gender to further explore their relationship. The results showed a specific non-linear relationship and saturation effect of BMI (kg/m^2^) with 3-month unfavorable outcomes in male patients but not in female patients. The inflection point for BMI was 23.35 kg/m^2^. When BMI was below 23.35 kg/m^2^ in men with AIS, BMI was inversely associated with unfavorable outcomes (OR=0.89,95% CI:0.80-0.98).

**Conclusion:**

A particular non-linear connection and saturation effect between BMI (kg/m^2^) and 3-month unfavorable outcomes were present in male patients with AIS but not in females. 23.35 kg/m^2^ was the BMI’s inflection point. The probability of unfavorable outcomes was substantially and inversely associated with BMI in men with AIS when it was less than 23.35 kg/m^2^. Men with AIS should have a BMI of no less than 23.35 kg/m^2^ to reduce the probability of unfavorable outcomes following AIS.

## Introduction

Ischemic stroke remains a leading cause of death and disability worldwide. It causes individuals and society as a whole to bear a heavy financial, clinical, and social cost (1–3). The 3-month functional outcome after an acute stroke event, as determined by the modified Rankin Scale (mRS), has been widely considered a prognosis indicator in stroke patients (4). An unfavorable outcome is generally defined as an mRS score ≥3, including functional dependence (moderate disability, moderately severe disability, severe disability) and death (5, 6). Previous studies have shown that 40% of patients with acute ischemic stroke(AIS) have an unfavorable outcome despite the best pharmacological treatment (7, 8). Accurate functional outcome prediction in stroke patients can improve clinical treatment, help with patient and family education and counseling, and simplify recovery and discharge planning (9). Therefore, finding predictive markers in stroke patients would be very helpful for risk stratification, therapy selection, and prognosis evaluation. Age, the cause of the stroke, the etiology of the stroke, obesity, diabetes, heart disease, and hypertension are the main known prognostic factors for stroke (3, 10–13).

Obesity is an established risk factor for stroke, and prevention guidelines recommend weight loss (14). However, the association between obesity and the prognosis of stroke victims is contentious and complicated. Several studies have reported a negative association between obesity and unfavorable clinical outcomes of ischemic stroke, such as stroke recurrence, mortality, and readmission (15–18). But others have found that in patients with ischemic stroke, being overweight or obese is not associated with lower mortality or better functional recovery, and the relationship between body mass index (BMI) and prognosis in stroke patients is not well established (19–21). This controversy has raised doubts regarding the usefulness of suggesting weight loss as secondary prophylaxis for stroke in people who are overweight or obese. Therefore, it remains quite important to explore the true relationship between BMI and the prognosis of acute ischemic stroke.

In addition, it is worth noting that most studies have explored the relationship between BMI and stroke prognosis mainly based on linear regression, and no studies have explored their non-linear association. Furthermore, considering the differences in body fat percentage and distribution patterns between men and women, their relationship may differ across genders. And unfortunately, no studies have presented the relationship between BMI and stroke prognosis in different genders. Therefore, the present study was designed to examine the linear and non-linear relationship between BMI and unfavorable outcomes in patients with AIS using the published data from a cohort study in South Korea.

## Methods

### Study design

This cohort research was done utilizing data from January 2010 to December 2016 from a single-center prospective registry system in South Korea (22). BMI was the relevant independent variable in this study. The dependent variable was the 3-month unfavorable outcome among AIS patients (dichotomous variable: unfavorable outcome, favorable outcome).

### Data source

The information was derived from this study: Kang MK, Kim TJ, Kim Y, et al: Geriatric nutritional risk index predicts poor outcomes in patients with acute ischemic stroke-automated undernutrition screen tool. PLoS ONE15 (2):e0228738. https://doi.org/10.1371/journal.pone.0228738 (22). This is an open-access article published in accordance with the Creative Commons Attribution License, which enables unlimited use, distribution, and reproduction in any form, as long as the original author and source are acknowledged (22). Here, we would like to express our gratitude to the authors for providing the data.

### Study population

The original researchers gathered individuals with AIS admitted to the hospital within 7 days after symptom onset using data from a prospective single-center registry (22). The original research was done with the consent of the Institutional Review Board at Seoul National University Hospital. In addition, the Institutional Review Board waived the necessity for patient consent (IRB No. 1009-062-332) (22). Therefore, ethical approval was not required for this secondary analysis. In addition, the original study was done in accordance with the Declaration of Helsinki; all methods were carried out in compliance with the pertinent standards and regulations, which include declarations in the Declarations section (22). So did this secondary analysis.

**Figure 1:**
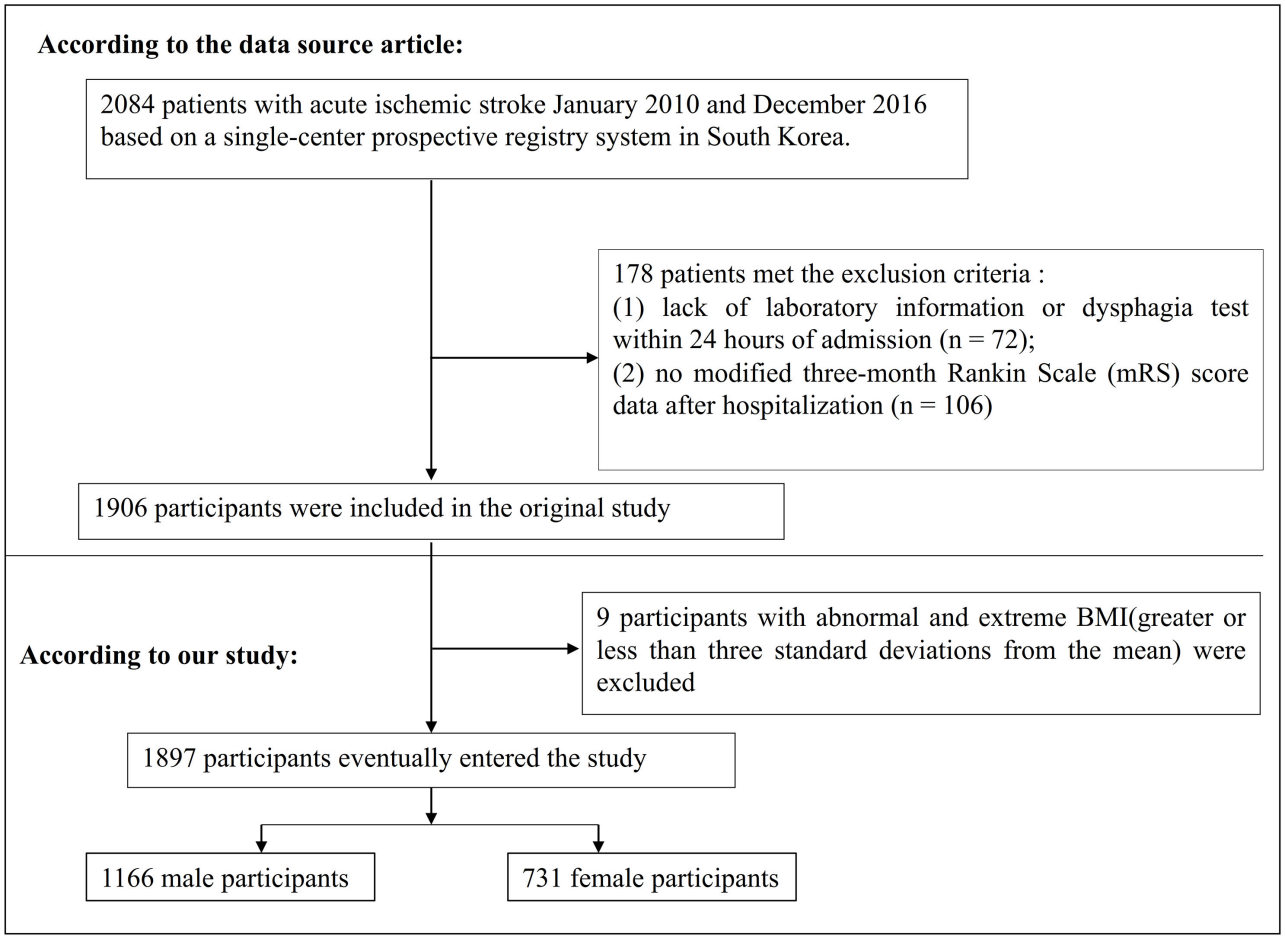
Flowchart of study participants.

The original study enrolled 2,084 patients with AIS. The types of AIS included large artery atherosclerosis, small vessel occlusion, cardioembolism, and other determined(transient ischemic attack, etc.). 178 participants meeting the exclusion criteria were excluded. The following were the original study’s exclusion criteria: (i) lack of dysphagia test or laboratory information within 24 hours after admission (n = 72); (ii) no modified three-month Rankin Scale (mRS) score after hospitalization (n = 106). Finally, the analysis of the initial study comprised a total of 1906 individuals (22). Participants with abnormal and excessive BMI (three standard deviations above or below three standard deviations from the mean) were not included in the current study(n = 9). Ultimately, the current study included a total of 1897 individuals with AIS. The procedure for selecting participants is shown in **Figure 1**.

### Variables

#### BMI

BMI was recorded as a continuous variable. The detailed procedure for defining BMI was as follows: BMI = weight (kg)/height^2^(m^2^). It was important to note that relevant information for height and weight was obtained at baseline. According to the WHO standards for the Asian population, the individuals were categorized as underweight(BMI < 18.5 kg/m^2^), normal (18.5 ≤ BMI < 23.0 kg/m^2^), overweight (23.0 ≤ BMI < 25.0 kg/m^2^), and obese (BMI ≥ 25.0 kg/m^2^) (23).

#### Three-month outcomes in individuals with acute ischemic stroke

The modified Rankin Scale (mRS) score was used to assess 3-month outcomes after the onset of AIS (22). Information was gathered by telephone or outpatient structured interviews (22). Participants were separated into two groups: those with favorable outcomes and those with unfavorable outcomes. Unfavorable results were classified as mRS scores≥3 (22, 24).

#### Covariates

Covariates were chosen in accordance with the prior research and our clinical expertise. The following variables were utilized as covariates: (i) categorical variables: sex, age, diabetes mellitus (DM), previous stroke/transient ischemic attack (TIA), atrial fibrillation, coronary heart disease (CHD), smoking status, hypertension, stroke etiology; (ii) continuous variables: total serum cholesterol (TC), hematocrit (HCT), serum high-density lipoprotein cholesterol (HDL-c), hemoglobin concentration (HGB), platelet (PLT), blood Urea Nitrogen (BUN), serum triglyceride (TG), serum low-density lipoproteins cholesterol (LDL-c), aspartate aminotransferase (AST), hemoglobin A1c (HBA1c), alanine aminotransferase (ALT), serum albumin (ALB), fibrinogen(FIB), serum creatinine (Scr), national institute of health stroke scale (NIHSS) score (2); Total cholesterol ≥ 6.2 mmol/L is considered hypercholesterolemia (25).

#### Data collection

Information related to data collection was obtained from the original study (22). Skilled nurses measured the patient’s height and weight on admission using an automatic scale (model GL-150, G-Tech International, Uijeongbu-si, Gyeonggi-do, South Korea). For patients with severe stroke who were unable to stand independently, weight was measured using an under-bed scale and height was measured using a tape measure. Laboratory information such as HGB, TG, TC, HDL-c, AST, ALT, ALB, LDL-c, HBA1C, BUN, and Scr was collected from electronic medical records. The National Institutes of Health Stroke Scale (NIHSS) score was assessed at admission to assess initial neurological severity. In addition, stroke subtypes were classified according to the Trial of Org 10172 in Acute Stroke Treatment (TOAST) (22).

#### Missing data handling

In our study, there were 1 (0.05%), 75 (3.95%), 1 (0.05%), 99 (5.23%), 19 (1%), 107 (5.64%), and 381 (20.08%) subjects with missing data for TC, LDL-c, AST, HDL-c, FIB, TG, and HBA1c, respectively. Multiple imputations are utilized for the missing data in this study to limit the deviation brought on by missing variables, which cannot accurately reflect the statistical effectiveness of the target sample throughout the modeling phase (26, 27). Age, sex, HGB, LDL-c, TG, HCT, AST, ALT, BUN, HBA1C, ALB, FIB, DM, hypertension, prior stroke or TIA, stroke etiology, atrial fibrillation, smoking, and NIHSS score were all included in the imputation model (the type was linear regression, and iterations were 10). Missing-at-random (MAR) assumptions are used in missing data analysis processes (27). It is important to note that only the variables TC, LDL-c, AST, HDL-c, FIB, TG, and HBA1c were imputed when the imputed data were pooled for analysis.

### Statistical analysis

The means and standard deviations were presented for continuous variables with Gaussian distributions, medians were reported for skewed distributions, and frequencies and percentages were presented for categorical variables. We used the Kruskal-Wallis H test (skewed distribution), the One-Way ANOVA test (normal distribution), or χ2 (categorical variables) to test for differences among different BMI groups.

Following covariate screening, we built three distinct models using univariate and multivariate binary logistic regression models to investigate the relationship between BMI and unfavorable outcomes following AIS in all male and female participants. The models were as follows:(i)a non-adjusted model (no covariates were adjusted); (ii)a minimally-adjusted model (Model I: adjusted for age, sex, smoking, NIHSS score); (iii)a fully-adjusted model (Model II: adjusted for sex, age, LDL-c, HGB, TG, AST, HCT, ALT, BUN, HBA1C, FIB, ALB, hypertension, DM, stroke etiology, previous stroke or TIA, smoking, atrial fibrillation, and NIHSS score). Effect sizes were calculated and reported with 95% confidence intervals (95%CI). Based on clinical knowledge, reports in the literature, and the findings of univariate analysis, we made adjustments for confounding variables (9, 28–30). It should be noted that in the male and female subgroups, the variables adjusted for in Model I and Model II do not include sex. Additionally, the final multivariate logistic regression equation excluded TC since it was collinear with other factors (**Supplementary Table S1**). We also applied a marginal structural model to explore the relationship between BMI and 3-month unfavorable outcomes in patients with acute ischemic stroke.

Using generalized additive models (GAM) and smooth curve fitting (penalized splines), we further investigated the non-linear association between BMI and unfavorable outcomes in the male, female, and all participants, respectively. If a non-linear relationship was found, we first used a recursive algorithm to find the inflection point. The recursive algorithm starts with a random initialization and then uses a filtering/smoothing step to find the inflection point. We then built a binary logistic regression model on both sides of the inflection point (31). The log-likelihood ratio test was used to find the best model to describe the link between BMI and unfavorable outcomes in participants with AIS.

We carried out several sensitivity analyses to evaluate the robustness of our findings. We transformed BMI into a categorical variable based on the World Health Organization’s definition of Asian populations. We estimated P for the trend to confirm the findings for BMI as a continuous variable and to investigate the potential for a non-linear link between BMI and 3-month unfavorable outcomes (the method used was a linear-by-linear association). Since smoking and hypercholesterolemia are strongly linked to unfavorable outcomes in AIS patients (32, 33), we excluded patients who smoked and had TC ≥6.2 mmol/L for sensitivity analyses to explore the association between BMI and unfavorable outcomes of AIS in men, women, and the entire sample, respectively. In addition, by computing E values, we investigated the potential for unobserved confounding between BMI and negative outcomes (34).

STROBE guidelines were followed in the writing of all findings (35). To conduct the necessary statistical tests, we utilized both Empower Stats (X&Y Solutions, Inc., Boston, MA, http://www.empowerstats.com) and R (http://www.r-project.org, The R Foundation). The cutoff for statistical significance was a P value of 0.05 (two-sided).

## Results

### Characteristics of participants

**Figure 2:**
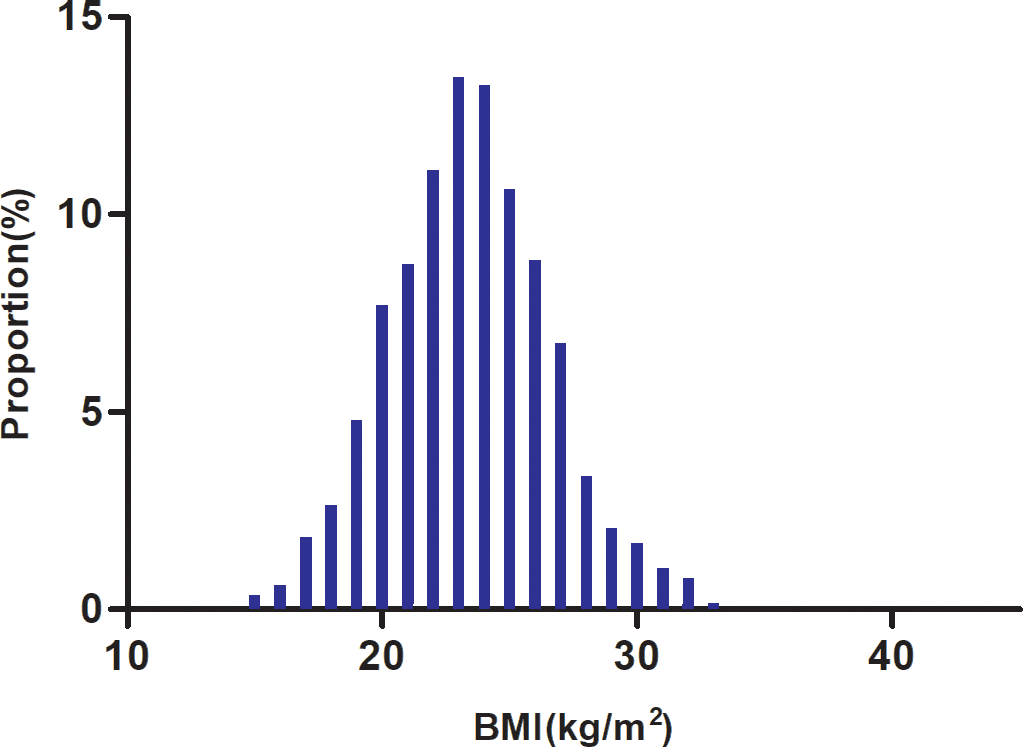
Distribution of BMI. It presented a normal distribution, ranging from 14.57 to 33.06 kg/m^2^, with a mean of 23.44 kg/m^2^.

**Table 1** details the demographic and clinical characteristics of the study’s participants. The final analysis comprised 1897 individuals, 61.47% of whom were men. There were 431 (22.72%), 503 (26.52%), 668 (35.21%), and 295 (15.55%) individuals who were under 60, between 60 and 70, between 70 and 80, and above 80 years old, respectively. BMI presents a normal distribution, ranging from 14.57 to 33.06kg/m^2^, with a mean of 23.44kg/m^2^ (**Figure 2**). NIHSS scores showed a skewed distribution ranging from 0 to 33 with a median (interquartile) of 3 (1, 7) (**Supplementary Figure S1**). We assigned adults into subgroups based on the WHO definitions for Asian populations for BMI categories. Compared with the underweight group, HGB, HCT, TC, TG, LDL-c, Scr, ALB, ALT, and HBA1c increased significantly in the obesity group, whereas the opposite results were found in the HDL-c, FIB, and NIHSS scores. In addition, the proportion of women, smoking status, CHD, and DM was lower in the obesity group. Furthermore, the stroke etiologies were more likely to be large artery atherosclerosis or cardio embolism in the obesity group.

**Table 1:** The baseline characteristics of participants.

BMI group (kg/m^2^)	Underweight (<18.5)	Normal (18.5-23)	Overweight (23-25)	Obesity (>=25)	*P*
**Participants**	104	742	493	558	
**Sex, n(%)**					<0.001
**Male**	55 (52.88%)	420 (56.60%)	332 (67.34%)	359 (64.34%)	
**Female**	49 (47.12%)	322 (43.40%)	161 (32.66%)	199 (35.66%)	
**Age(years), n(%)**					<0.001
**<60**	12 (11.54%)	143 (19.27%)	103 (20.89%)	173 (31.00%)	
**60 to <70**	19 (18.27%)	178 (23.99%)	149 (30.22%)	157 (28.14%)	
**70 to <80**	43 (41.35%)	277 (37.33%)	171 (34.69%)	177 (31.72%)	
**≥80**	30 (28.85%)	144 (19.41%)	70 (14.20%)	51 (9.14%)	
**HGB(g/dL),mean ± sd**	11.98 ± 2.03	13.09 ± 1.99	13.63 ± 1.80	14.13 ± 1.92	<0.001
**HCT (%),mean ± sd**	36.09 ± 5.78	39.00 ± 5.57	40.51 ± 5.03	41.83 ± 5.36	<0.001
**PLT (10^9/L), mean ± sd**	227.00 ± 78.79	221.98 ± 74.38	220.19 ± 68.38	228.11 ± 67.92	0.264
**TC (mmol/L), mean ± sd**	4.33 ± 1.04	4.61 ± 1.09	4.59 ± 1.11	4.76 ± 1.18	<0.001
**TG (mmol/L), mean ± sd**	1.01 ± 0.44	1.14 ± 0.55	1.29 ± 0.62	1.43 ± 0.74	<0.001
**HDL-c(mmol/L), mean ± sd**	1.25 ± 0.43	1.25 ± 0.38	1.18 ± 0.32	1.15 ± 0.31	<0.001
**LDL-c(mmol/L), mean ± sd**	2.57 ± 0.86	2.74 ± 0.90	2.78 ± 0.98	2.88 ± 1.03	0.006
**BUN (mg/dl),median (quartile)**	15.00 (12.00-22.00)	15.00 (12.00-20.00)	17.00 (13.00-21.00)	15.00 (13.00-19.00)	0.003
**Scr(mg/dl), median (quartile)**	0.85 (0.67-1.13)	15.00 (12.00-20.00)	0.91 (0.76-1.12)	0.92 (0.76-1.08)	<0.001
**AST(U/L), median (quartile)**	24.00 (19.75-31.00)	22.00 (18.00-29.00)	22.00 (18.00-28.00)	24.00 (19.00-30.75)	<0.001
**ALT(U/L), median (quartile)**	15.00 (11.00-21.00)	17.00 (12.00-22.00)	18.00 (14.00-26.00)	21.00 (15.00-32.00)	<0.001
**ALB(g/dL), mean ± sd**	3.77 ± 0.54	3.97 ± 0.45	4.05 ± 0.36	4.10 ± 0.40	<0.001
**HBA1c(%), mean ± sd**	6.14 ± 1.02	6.26 ± 1.07	6.28 ± 1.11	6.37 ± 1.19	0.147
FIB (m ***g/L***), **mean ± sd**	360.32 ± 116.43	337.72 ± 87.87	330.21 ± 81.31	327.00 ± 81.16	0.001
**NIHSS score, median (quartile)**	5.00 (2.00-11.00)	4.00 (1.25-8.75)	3.00 (1.00-7.00)	3.00 (1.00-6.00)	<0.001
Previous stroke/TIA, **n(%)**	20 (19.23%)	155 (20.89%)	119 (24.14%)	108 (19.35%)	0.265
**Hypertension, n(%)**	55 (52.88%)	433 (58.36%)	323 (65.52%)	394 (70.61%)	<0.001
**Diabetes**	25 (24.04%)	242 (32.61%)	162 (32.86%)	181 (32.44%)	0.341
**Smoking**	30 (28.85%)	283 (38.14%)	200 (40.57%)	235 (42.11%)	0.062
**Atrial fibrillation, n(%)**	31 (29.81%)	159 (21.43%)	97 (19.68%)	119 (21.33%)	0.155
**CHD, n(%)**		81 (10.92%)	57 (11.56%)	75 (13.44%)	0.205
**Stroke etiology, n(%)**					0.136
**SVO**	14 (13.46%)	131 (17.65%)	96 (19.47%)	124 (22.22%)	
**LAA**	26 (25.00%)	231 (31.13%)	163 (33.06%)	181 (32.44%)	
**CE**	31 (29.81%)	197 (26.55%)	124 (25.15%)	140 (25.09%)	
**Other determined**	11 (10.58%)	79 (10.65%)	40 (8.11%)	40 (7.17%)	
**Undetermined**	22 (21.15%)	104 (14.02%)	70 (14.20%)	73 (13.08%)	

SD, standard deviation; n, number.

HGB, hemoglobin concentration; PLT, platelet; HCT, hematocrit; TC, total cholesterol; TG, triglyceride; LDL-c, low-density lipoproteins cholesterol; HDL-c, high-density lipoprotein cholesterol; Scr, serum creatinine; BUN, blood urea nitrogen; AST, aspartate aminotransferase; ALT, alanine aminotransferase; ALB, serum albumin; BMI, body mass index; FIB, fibrinogen; CHD, coronary heart disease; large artery atherosclerosis; SVO, small vessel occlusion;TIA, transient ischemia attack; LAA, small vessel occlusion; NIHSS, national institute of health stroke scale; CE, cardio embolism.

### The prevalence rate of unfavorable outcomes three months following acute ischemic stroke

**Figure 3:**
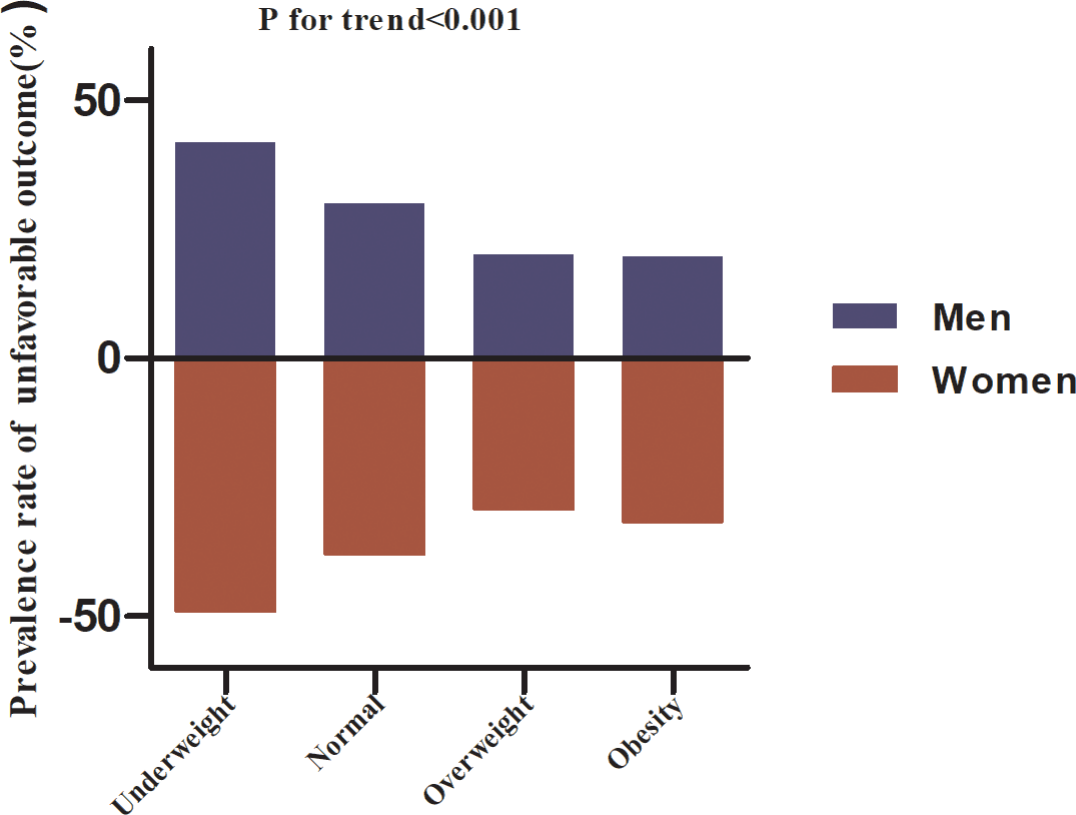
The prevalence of unfavorable outcomes in men and women according to BMI classification.

**Table 2** showed that a total of 543 participants had an unfavorable outcome. The overall prevalence of unfavorable outcomes was 28.62%. Specifically, the prevalence of unfavorable outcomes in the underweight, normal, overweight, and obesity groups was 45.19%, 33.42%, 23.12%, and 24.01%, respectively. The prevalence of unfavorable outcomes was lower in the overweight and obesity groups compared to the underweight group, and the prevalence of unfavorable outcomes was similar in the overweight and obesity groups. The overall prevalence of unfavorable outcomes was 24.61% in men. Specifically, in men, the prevalence of unfavorable outcomes was 41.82%, 30%, 20.18%, and 19.78% in the underweight, normal, overweight, and obese groups, respectively. In addition, the overall prevalence of unfavorable outcomes in female AIS patients was 24.61%. The prevalence of unfavorable outcomes in women with AIS was 41.82%, 30%, 20.18%, and 19.78% in the underweight, normal, overweight, and obesity groups, respectively (**Table 2**; **Figure 3**).

**Table 2:** Prevalence rate of unfavorable outcome (mRS score≥3) 3-month after stroke in men, women and all population.

BMI	Participants	participants with unfavorable outcomes	Prevalence rate (95% CI) (%)
**Men**			
Total	1166	287	24.61 (22.14-27.09)
Normal	55	23	41.82 (28.36-55.28)
Underweight	420	126	30.00 (25.60-34.40)
Overweight	332	67	20.18 (16.30-25.19)
Obesity	359	71	19.78 (15.64-23.92)
*P* for trend			<0.001
**Women**			
Total	731	256	35.02 (31.55-38.49)
Normal	49	24	48.98 (34.47-63.49)
Underweight	322	122	48.98 (34.47-63.49)
Overweight	161	47	29.19 (22.09-36.29)
Obesity	199	63	31.66 (25.14-38.18)
*P* for trend			<0.001
**All participants**			
Total	1897	543	28.62 (26.59-30.66)
Normal	104	47	45.19 (35.47-54.92)
Underweight	742	248	33.42 (30.02-36.83)
Overweight	493	114	23.12 (19.39-26.86)
Obesity	558	134	24.01 (20.46-27.57)
*P* for trend			<0.001

BMI body mass index; mRS, modified Rankin scale.

**Figure 4:**
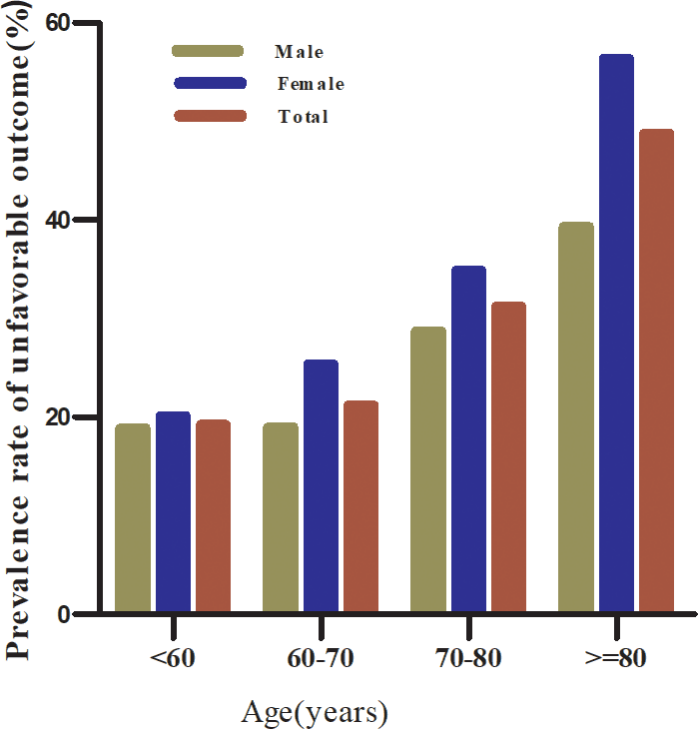
The prevalence of unfavorable outcomes among participants according to age stratification.

In the age stratification based on age <60, 60 to <70, 70 to <80, and ≥80, the prevalence of unfavorable outcomes among participants with AIS was higher in women than in men, regardless of age group. Incidence was also found to increase with age in both males and females (**Figure 4**).

### The results of univariate analyses using a binary logistic regression model

The results of the univariate analyses demonstrated that there was no association between the risk of unfavorable outcomes in AIS patients and PLT (OR=0.999, 95%CI:0.998-1.001), HDL-c (odds ratio OR=0.881, 95%CI: 0.662-1.173), Scr (OR=1.017, 95%CI:0.927-1.117), or CHD (OR=1.026, 95%CI:0.753-1.398) (all P>0.05), but was positively associated with FIB (OR=1.003, 95%CI:1.002-1.004) and BUN (OR=1.016, 95%CI:1.006-1.027), (all P<0.05). In addition, patients with age ≥80 years old (OR=3.998, 95%CI:2.872-5.556), hypertension (OR=1.345, 95%CI:1.103-1.716), diabetes (OR=1.437, 95%CI:1.167-1.771), previous stroke/TIA (OR=1.345, 95%CI:1.089-1.661), and other determined of stroke etiology were more likely to experience unfavorable outcomes (all P<0.05). However, HCT (OR=0.933, 95%CI:0.916-0.950), HGB (OR=0.821, 95%CI:0.662-1.173), BMI (OR=0.908, 95%CI:0.879-0.938), AST (OR=0.993, 95%CI:0.986-1.000), ALB (OR=0.272, 95%CI:0.213-0.347), TC (OR=0.835, 95%CI:0.762-0.916), and TG (OR=0.995, 95%CI:0.993-0.998) was negatively related to the probability of unfavorable outcomes(**Supplementary Table 2**).

### Results of multivariate analysis using binary logistic regression models

Three models were constructed using binary logistic regression models to examine the association between BMI and the risk of unfavorable outcomes after AIS in all participants, male and female.

First, in the crude model, a 1-unit increase in BMI was associated with a 9.2% reduction in the probability of unfavorable outcomes (OR=0.908, 95% CI 0.879-938, P<0.001). However, the multiple regression analysis results were not statistically significant in both the minimally adjusted and fully adjusted models (P>0.05). In addition, the results of the multiple regression analysis based on gender stratification were similarly not statistically significant (P>0.05) (**Table 3**).

**Table 3:** Relationship between BMI and 3-month unfavorable outcomes in patients with acute ischemic stroke in different models.

Exposure		Crude model (OR,95%CI) *P*	Model I(OR,95%CI) *P*	Model II(OR,95%CI) *P*
**All**	BMI (kg/m^2^)	0.908 (0.879, 0.938) <0.001	0.960 (0.924, 0.998) 0.037	0.966 (0.926, 1.008) 0.107
	BMI group			
	Normal	Ref	Ref	Ref
	Underweight	1.642 (1.084, 2.488) 0.019	1.285 (0.784, 2.105) 0.319	1.233 (0.729, 2.084) 0.435
	Overweight	0.599 (0.462, 0.776) <0.001	0.698 (0.515, 0.945) 0.020	0.722 (0.526, 0.990) 0.043
	Obesity	0.630 (0.492, 0.806) <0.001	0.877 (0.655, 1.175) 0.381	0.957 (0.698, 1.311) 0.785
**Male**	BMI (kg/m^2^)	0.894 (0.854, 0.936) <0.001	0.951 (0.903, 1.003) 0.062	0.980 (0.924, 1.040) 0.505
	BMI group			
	Normal	Ref	Ref	Ref
	Underweight	1.677 (0.944, 2.980) 0.078	1.287 (0.656, 2.527) 0.463	1.108 (0.519, 2.364) 0.791
	Overweight	0.590 (0.420, 0.829) 0.003	0.666 (0.452, 0.982) 0.040	0.738 (0.491, 1.109) 0.144
	Obesity	0.575 (0.412, 0.803) 0.001	0.814 (0.555, 1.194) 0.293	0.989 (0.651, 1.504) 0.960
**Female**	BMI (kg/m^2^)	0.933 (0.890, 0.978) 0.004	0.966 (0.913, 1.023) 0.235	0.952 (0.893, 1.013) 0.122
	BMI group			
	Normal	Ref	Ref	Ref
	Underweight	1.574 (0.861, 2.878) 0.141	1.280 (0.616, 2.661) 0.508	1.367 (0.627, 2.979) 0.432
	Overweight	0.676 (0.450, 1.016) 0.060	0.720 (0.441, 1.175) 0.189	0.694 (0.409, 1.179) 0.177
	Obesity	0.759 (0.522, 1.104) 0.149	0.951 (0.600, 1.509) 0.833	0.927 (0.563, 1.527) 0.766

Crude mode1: we did not adjust other covariates

Model I: we adjusted age, sex, smoking, NIHSS score.

Model II: we adjusted age, sex, LDL-c, HGB, TG, AST, HCT, ALT, BUN, HBA1C, FIB, ALB, previous stroke or TIA, atrial fibrillation, hypertension, smoking, DM, stroke etiology, and NIHSS score.

In the male and female subgroups, Models I and II were not adjusted for the stratification variable sex.

Besides, we converted BMI from a continuous variable to a categorical variable and then reintroduced the categorically transformed BMI into the model. The results based on the multivariate-adjusted model for all participants showed an OR of 1.233 (0.729, 2.084) for underweight participants, 0.722 (0.526, 0.990) for overweight participants, and 0.957 (0.698, 1.311) for obese participants using participants with normal BMI as a reference.The distribution of confidence intervals showed that there was no statistically significant relationship between BMI (classification) derived from the model and the probablity of unfavorable outcomes following AIS (**Table 3**). Similar outcomes were found for multivariate-adjusted models based on AIS patients who were either male or female.

### The generalized additive model addressed the non-linear relationship between BMI and unfavorable outcomes

**Figure 5:**
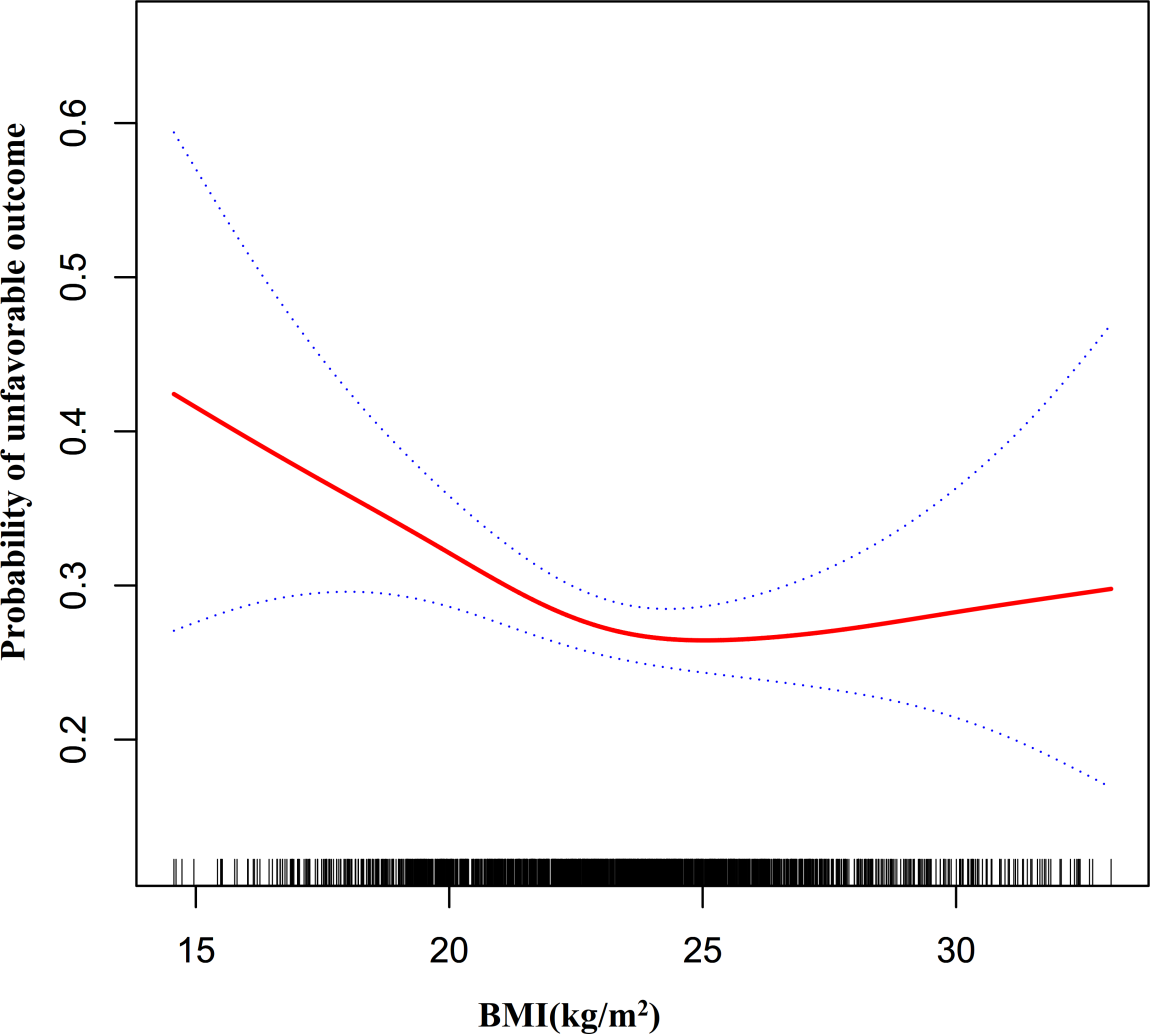
The non-linear relationship between BMI and the probability of unfavorable outcomes in all participants.. A non-linear relationship was detected in men with AIS after adjusting for age, LDL-c, HGB, TG, AST, HCT, ALT, BUN, HBA1C, FIB, ALB, previous stroke or TIA, atrial fibrillation, hypertension, smoking, DM, stroke etiology, and NIHSS score. In contrast, in women with AIS, the non-linear relationship between BMI and the risk of unfavorable outcomes in patients with AIS did not establish.

We further explored whether there was a non-linear association between BMI and unfavorable outcomes in patients with AIS. We discovered a non-linear relationship between BMI and risk of unfavorable outcomes in AIS patients using the GAM and smooth curve fitting (adjusted sex, age, LDL-c, HGB, TG, HCT, AST, ALT, BUN, HBA1C, FIB, ALB, hypertension, stroke etiology, atrial fibrillation, previous stroke or TIA, smoking status, DM, and NIHSS score) (**Figure 5**). Because of this, we fitted data to a piecewise binary logistic regression model to fit two distinct slopes. We also used a typical binary logistic regression model to fit the data, and we used the log-likelihood ratio test to determine which model had the greatest fit. The log-likelihood ratio test’s P value in our investigation was less than 0.05. By employing a recursive algorithm, we first determined the BMI inflection point, which was 23.07 kg/m^2^, and then we used a two-piecewise binary logistic regression model to evaluate the effect sizes and confidence intervals on the left and right of the inflection point. A 12% reduced probability of unfavorable outcomes was linked to each unit rise in BMI on the left side of the inflection point (OR= 0.88, 95%CI:0.82 to 0.96, p = 0.003). The effect size (OR) on the right side of the inflection point was 1.04 (95%CI:0.97-1.12, p = 0.27) (**Table 4**).

**Table 4:** The results of two-piecewise linear regression model.

Unfavorable outcome:	All participants (OR,95%CI, *P*)	Male (OR,95%CI, *P*)	Female (OR,95%CI, *P*)
Fitting model by standard linear regression	0.97 (0.926, 1.008) 0.107	0.98 (0.92, 1.04) 0.505	0.95 (0.89, 1.01) 0.122
Fitting model by two-piecewise linear regression			
Inflection point of BMI (kg/m^2^)	23.07	23.35	22.93
≤ Inflection point	0.88 (0.82, 0.96) 0.003	0.89 (0.80, 0.98) 0.023	0.89 (0.79, 1.01) 0.077
> Inflection point	1.04 (0.97, 1.12) 0.270	1.08 (0.98, 1.20) 0.127	1.00 (0.90, 1.11) 0.989
*P* for log-likelihood ratio test	0.011	0.022	0.251

In all participants, we adjusted age, sex, LDL-c, HGB, TG, AST, HCT, ALT, BUN, HBA1C, FIB, ALB, previous stroke or TIA, atrial fibrillation, hypertension, smoking, DM, stroke etiology, and NIHSS score.

For male and female subgroups, we adjusted for age, HGB, LDL-c, TG, HCT, AST, ALT, BUN, HBA1C, ALB, FIB, DM, previous stroke or TIA, hypertension, stroke etiology, atrial fibrillation, smoking, and NIHSS score.

**Figure 6:**
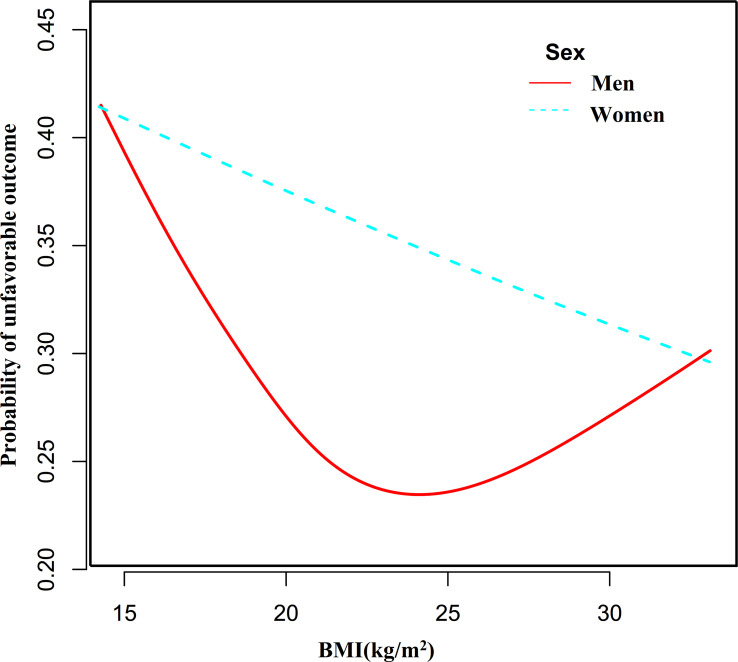
The nonlinear relationship between BMI and the probability of unfavorable outcomes in men and women.

By the same method, we found that the association between BMI and the probability of unfavorable outcomes in men with AIS was also non-linear (**Figure 6**, P for the log-likelihood ratio test <0.05). The inflection point for BMI was 23.35 kg/m^2^ and on the left side of the inflection point, each 1-unit increase in BMI was associated with an 11% reduction in the risk of unfavorable outcomes (OR= 0.89, 95% CI: 0.80 to 0.98, P=0.023). Their relationship was not statistically significant on the right side of the inflection point. In contrast, the non-linear association between BMI and the probability of unfavorable outcomes was not established in women with AIS (P for the log-likelihood ratio test was greater than 0.05).

### Sensitivity analysis

We conducted a series of sensitivity analyses to ensure our findings were robust. First, we applied the marginal structural model to explore the relationship between BMI and unfavorable outcomes in acute ischemic stroke patients in the overall population, male and female participants and found no statistically significant linear association between them (all p>0.05) (**Supplementary Table S3**). In addition, we performed sensitivity analyses on nonsmoking participants (n=1,149). After adjusting for confounding variables (including age, HGB, LDL-c, TG, AST, HCT, ALT, BUN, HBA1C, FIB, ALB, previous stroke or TIA, hypertension, NIHSS score, DM, atrial fibrillation, and stroke etiology), The results of the two-segment linear regression model analysis showed a non-linear relationship between BMI and the probability of unfavorable outcomes in men with AIS, while neither a linear nor a non-linear relationship between them held in women with AIS. In sensitivity analyses, we also excluded participants with TC≥6.2 mmol/L (n=1,732). After adjusting for confounding variables (including age, HGB, LDL-c, TG, AST, HCT, ALT, BUN, HBA1C, FIB, ALB, previous stroke or TIA, hypertension, NIHSS score, DM, atrial fibrillation, smoking, and stroke etiology), similar results were obtained, i.e., the association between BMI and the probability of unfavorable outcomes was non-linear in men with AIS, whereas their relationship was not statistically significant in women with AIS (**Table 5**). We also calculated an E-value to assess sensitivity to unmeasured confounders. Unknown or unmeasured variables may have had little effect on the association between BMI and unfavorable outcomes in patients with AIS, as the E-value (1.28) was greater than the relative risk of BMI and unmeasured confounders (1.14). Based on all sensitivity analyses, it is clear that our findings are robust.

**Table 5:** Relationship between BMI and unfavorable outcome 3-month after stroke analyzed by two-piecewise linear regression model in different sensitivity analyses.

Unfavorable outcome:	Male (OR,95%CI, *P*)	Female (OR,95%CI, *P*)	Total (OR,95%CI, *P*)
**Model I:**			
Fitting model by two-piecewise linear regression			
Inflection point of BMI (kg/m^2^)	23.32	23.00	23.03
**≤** Inflection point	0.90 (0.80, 1.00) 0.052	0.88 (0.77, 1.00) 0.044	0.88 (0.81, 0.96) 0.004
**>** Inflection point	1.08 (0.97, 1.20) 0.171	1.02 (0.92, 1.14) 0.680	1.05 (0.97, 1.13) 0.214
*P* for log-likelihood ratio test	0.047	0.127	0.011
**Model II:**			
Fitting model by two-piecewise linear regression			
Inflection point of BMI (kg/m^2^)	23.19	22.90	22.99
**≤** Inflection point	0.75 (0.63, 0.90) 0.002	0.89 (0.78, 1.01) 0.071	0.85 (0.76, 0.94) 0.001
**>** Inflection point	1.04 (0.87, 1.25) 0.639	1.01 (0.91, 1.12) 0.888	1.01 (0.93, 1.11) 0.787
*P* for log-likelihood ratio test	0.034	0.206	0.027

Model I was a sensitivity analysis in participants without TC ≥6.20mmol/L(n=1732). We adjusted age, sex, LDL-c, HGB, TG, AST, HCT, ALT, BUN, HBA1C, FIB, ALB, previous stroke or TIA, atrial fibrillation, hypertension, smoking, DM,stroke etiology, and NIHSS score.

Model II was a sensitivity analysis conducted on non-smoking participants (n= 1149). We adjusted age, sex, LDL-c, HGB, TG, AST, HCT, ALT, BUN, HBA1C, FIB, ALB, previous stroke or TIA, atrial fibrillation, hypertension, DM,stroke etiology, and NIHSS score.

OR,odds ratios; CI, confidence; Ref: reference.

## Discussion

The current study was designed to examine the association between BMI and 3-month outcomes in patients with AIS. We found a non-linear rather than linear relationship between them in male patients with AIS. The relationship between BMI and 3-month unfavorable outcomes in men with AIS is a saturation effect curve with an inflection point of 23.35 kg/m^2^ of BMI. In contrast, neither linear nor non-linear associations between BMI and 3-month outcomes were found in women with AIS.

There have been many findings in the past suggesting that elevated BMI is associated with a higher risk of ischemic stroke (36–39). However, the relationship between BMI and the prognosis of stroke patients is uncertain and controversial. Some studies have found that there is an inverse relationship between BMI and functional outcomes and death, which they call the “obesity paradox” (19, 40). A prospective cohort study from Korea, including 1,592 stroke patients, showed a negative association between BMI levels and initial neurological function (21). Another study showed that the incidence of short-term unfavorable outcomes was significantly higher in patients with AIS who were underweight (BMI <18.5 kg/m^2^) than in participants with normal weight (BMI:18.5-25 kg/m^2^) after adjusting for confounders (OR=1.82, 95% CI: 1.09-3.04). In contrast, there was no significant difference in the results of obese AIS patients (BMI>25 kg/m^2^) compared with those with normal BMI (16). Similarly, another cohort study concluded that BMI was negatively associated with the risk of mortality in patients with AIS (41). Compared with AIS patients with normal BMI, underweight AIS patients had a 36% increased risk of mortality (HR=1.36, 95% CI: 1.04 to 1.79), whereas the risk of mortality in the obese patients was significantly reduced by 34% (HR=0.66, 95% CI: 0.54 to 0.81) (41).In contrast, a study from the United States concluded that BMI was not associated with clinical outcomes in stroke patients treated with intravenous thrombolysis (42). Similarly, another observational study did not observe any difference in functional outcomes between obese (BMI ≥ 30 kg/m^2^) and normal weight AIS patients (BMI 18.5-24.9 kg/m^2^), overweight (BMI 25.0-29.9 kg/m^2^) and normal weight AIS patients (43). These inconsistent results are caused by the following possible explanations: first, the sample sizes varied considerably between studies; second, these studies were adjusted for different covariates; and third, the populations studied were different. Our study is consistent with these studies in that a linear relationship between BMI and prognosis in patients with AIS does not hold. It is worth considering that the conclusions based on linear regression analysis may be influenced by non-linear relationships, resulting in differences in the fitted linear relationships. In other words, there may be a non-linear relationship between BMI and the probability of unfavorable outcomes in patients with AIS. In this study, we used logistic regression with a cubic spline function and a smoothed curve fit (cubic spline smoothing) to test our hypothesis. Ultimately, we observed that the association between BMI and the probability of unfavorable outcomes was non-linear. In addition, we obtained an inflection point of 23.07 kg/m^2^ for BMI and then calculated the OR and CI around the inflection point using a two-piecewise logistic regression model.

A non-linear relationship is another term for a relationship between two variables in which a change in one variable does not always result in a corresponding change in the other variable. This could imply that the relationship between these two variables appears erratic or hardly present. Although more complex than linear relationships, non-linear relationships between entities can nevertheless be quite predictable. The non-linear relationship may be a more accurate representation of the genuine association between BMI and unfavorable outcomes in patients with AIS due to the complexity of the relationship between them.

In addition, the non-linear relationship between men and women may vary due to differences in body fat percentage and distribution patterns. Therefore, we stratified AIS patients by sex to further explore the link between BMI and unfavorable outcomes. The relationship between BMI and 3-month outcome in male patients with AIS was found to be a saturated effect curve with an inflection point of 23.35 kg/m^2^ for BMI. In contrast, neither a linear nor a non-linear relationship was found between BMI and the probability of adverse outcomes in female AIS patients. This facilitates clinical consultation and provides a basis for decision-making to optimize stroke rehabilitation. In order not to increase the probability of unfavorable outcomes after AIS, men with AIS should have a BMI of no less than 23.35 kg/m^2^.

However, the reasons for the non-linear relationship between BMI and the probability of unfavorable outcomes in patients with AIS are unclear. First, a too-low BMI is an indicator of malnutrition and may affect neuro repair (44). In addition, AIS is a state of metabolic stress with high energy demands. Post-stroke neuroendocrine sympathetic activation, cytokine, and anaerobic radical accumulation lead to a catabolic/anabolic imbalance requiring the consumption of fat and muscle tissue (45). Therefore, low body weight is not conducive to early neurological repair in AIS patients. Conversely, excessive body weight reserves of energy and nutrients exceed the need for early neurological repair to reach saturation. Therefore, on the left side of the inflection point, the negative association between BMI and the probability of unfavorable outcomes may be due to insufficient nutritional and energy reserves affecting early neurological repair. On the right side of the inflection point, the relationship is not statistically significant, possibly because patients with excess BMI possess more energy and nutrients than are needed for neurological repair, and the probability of unfavorable outcomes does not decrease with further increases in BMI (44). Moreover, males and females have different proportions and distributions of body fat, which may explain the differences in the association between BMI and the probability of unfavorable outcomes between men and women in our findings (46).

We enumerated the following strengths of our study. First, the independent variables in our study employed both categorical and continuous BMI to evaluate its association with the negative result of AIS, reducing information loss and quantifying their relationship. Most covariates have full information, and only a few are missing information. Second, Missing data were handled using multiple imputations. This approach can enhance statistical power and reduce potential bias brought on by the absence of covariate information. Third, our study is a significant improvement compared to previous studies regarding the nonlinearity addressing. In addition, we ran a battery of sensitivity analyses to ensure the stability of the outcomes(reanalyzing the non-linear correlation between BMI and unfavorable outcomes of AIS after omitting participants who smoked and had TC > 6.2 mmol/L and calculating E-values to explore the potential for unmeasured confounding).

Potential drawbacks should be taken into account. First, Koreans make up the study’s sample population. The applicability of these results to other races needs further verification. Second, some of the variable data was lacking. For instance, instead of patient-specific age, the initial study database included age-stratified information in 10 intervals, which might lead to incomplete variable information. In the future, we may think about how to design our study, and we’ll get more precise variable data. The third drawback is that BMI was only calculated once at the time of admission. We thus don’t know if the BMI altered after 24 hours of admission. It’s a significant question that could call for further study. Fourth, as with all observational research, there may still have been unmeasured or uncontrolled confounders despite the fact that recognized potential confounders were controlled. Furthermore, because this study is a secondary analysis based on published data, factors not included in the data set, including details on intravenous thrombolysis or endovascular thrombectomy, cannot be adjusted. However, we estimated the E-value and discovered that unmeasured or uncontrolled confounders were unlikely to explain our findings.

## Conclusion

In patients with AIS, BMI (kg/m^2^) had a specific non-linear relationship and saturation effect with unfavorable 3-month outcomes in male patients but not in female patients. The inflection point of BMI was 23.35 kg/m^2^. When BMI was below 23.35 kg/m^2^ in men with AIS, BMI was significantly and negatively associated with the probability of unfavorable outcomes. When BMI was ≥23.35 kg/m2, their relationship was not statistically different. This helps to facilitate physician-patient communication and consultation and provides a basis for decision-making to optimize ischemic stroke rehabilitation. In order not to increase the probability of unfavorable outcomes after AIS, men with AIS should have a BMI of no less than 23.35 kg/m^2^.

## Data availability statement

The original contributions presented in the study are included in the article/**Supplementary Materials**. Further inquiries can be directed to the corresponding authors.

## Ethics statement

The studies involving human participants were reviewed and approved by Institutional Review Board of Seoul National University Hospital. Written informed consent for participation was not required for this study in accordance with the national legislation and the institutional requirements.

## Author contributions

YZ, WX, XZ, CC, and HH conceived the research, drafted the manuscript, and performed the statistical analysis. YW and HO revised the manuscript and designed the study. All authors read and approved the final manuscript.
