# Supplementary figures and images for: Body mass index has a non-linear association with three-month outcomes in men with acute ischemic stroke: An analysis based on data from a prospective cohort study
Source: Front Endocrinol (Lausanne). 2022 Dec 12;13:1041379. doi: 10.3389/fendo.2022.1041379 (PMC9792146; doi:10.3389/fendo.2022.1041379)

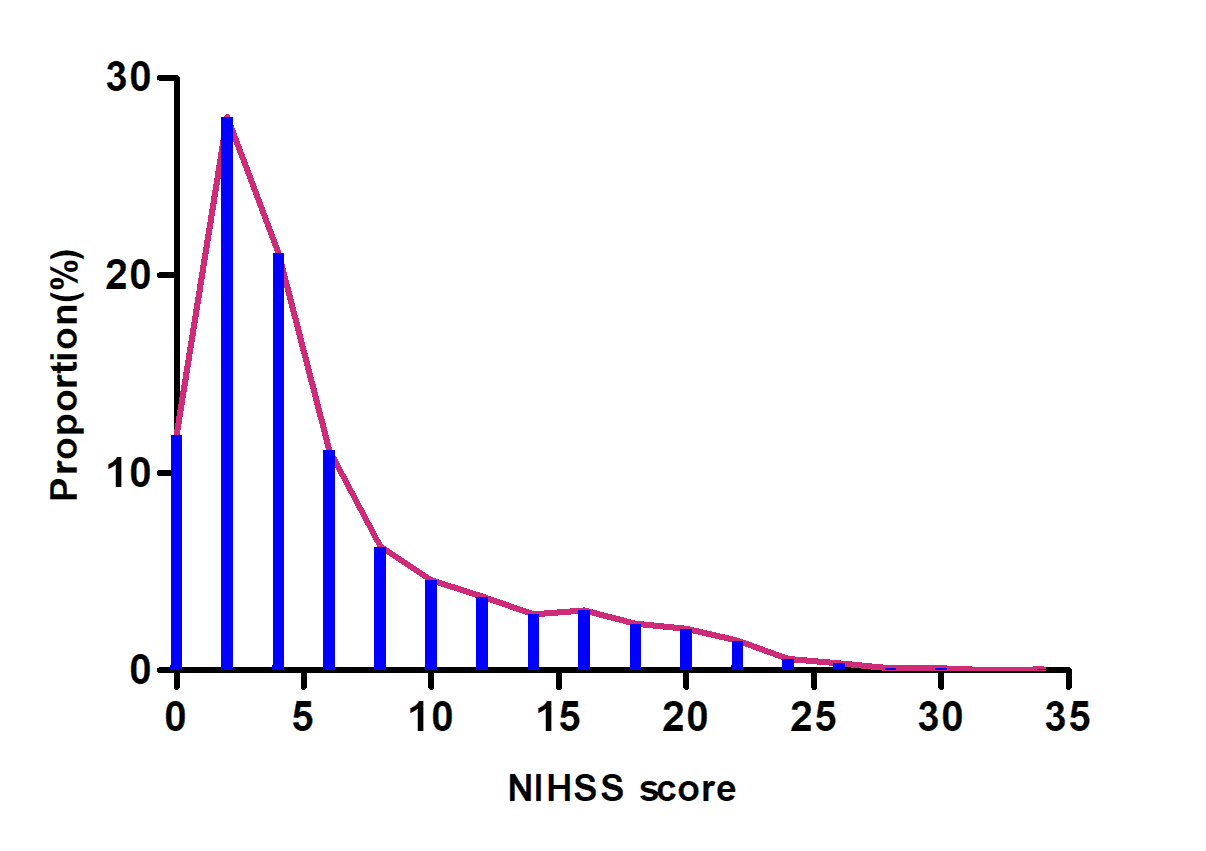

Supplement: Supplementary file 1 [file Image_1.tif]
