# Supplementary material for: Body mass index has a non-linear association with three-month outcomes in men with acute ischemic stroke: An analysis based on data from a prospective cohort study
Source: Front Endocrinol (Lausanne). 2022 Dec 12;13:1041379. doi: 10.3389/fendo.2022.1041379 (PMC9792146; doi:10.3389/fendo.2022.1041379)
Supplement: Supplementary file 2 [file DataSheet_1.docx]

**Body mass index has a non-linear association with three-month outcomes in men with acute ischemic stroke: an analysis based on data from a prospective cohort study**

**Running title:** **BMI and stroke outcome**

**Yibing Zan^1,2#^, Wei Xiong^3#^, Xiaohua Zhang^1^, Yong Han^4^, Changchun Cao^1^, Haofei Hu^5^, Yulong Wang^1^*, Haining Ou^2^***

^1^Department of Rehabilitation, Shenzhen Dapeng New District Nan'ao People's Hospital, Shenzhen 518000, Guangdong Province, China

^2^Department of Rehabilitation Medicine, The Fifth Affiliated Hospital of Guangzhou Medical University, Guangzhou, 510000, Guangdong Province, China

^3^Department of Rehabilitation, Shenzhen Second People's Hospital, The First Affiliated Hospital of Shenzhen University, Shenzhen 518000, Guangdong Province, China

^4^Department of Emergency, Shenzhen Second People's Hospital, Shenzhen 518000, Guangdong Province, China

^5^Department of Nephrology, Shenzhen Second People's Hospital, Shenzhen 518000, Guangdong Province, China

**^#^ Yibing Zan and Wei Xiong have contributed equally to this work.**

***Corresponding author**

Yulong Wang

Department of Rehabilitation,

Shenzhen Dapeng New District Nan'ao People's Hospital,

No. 6, Renmin Road, Dapeng New District,

Shenzhen 518000,

Guangdong Province, China

Tel: +86-755-83366388

E-mail: [992051016@qq.com](mailto:992051016@qq.com)

***Corresponding author**

Haining Ou

Department of Rehabilitation Medicine,

The Fifth Affiliated Hospital of Guangzhou Medical University,

No. 621 Gangwan Road, Huangpu Street, Huangpu District,

Guangzhou, 510000, Guangdong Province, China

Tel: +86-18156181316

E-mail: 705034851@qq.com

Table S1 Collinearity screening

|  | Step 1 | Step 2 | Step 3 |
| --- | --- | --- | --- |
| BMI(kg/m2) | 1.3 | 1.3 | 1.3 |
| HGB（g/dL） | 22.8 | 22.5 | 22.5 |
| HCT (%) | 22 | 1.8 | 1.8 |
| PLT (10^9/L) | 1.2 | 1.2 | 1.2 |
| TC (mmol/L) | 6.7 | 6.7 | NA |
| TG (mmol/L) | 1.6 | 1.6 | 1.3 |
| HDL-c(mmol/L) | 1.7 | 1.7 | 1.3 |
| LDL-c(mmol/L) | 5.3 | 5.3 | 1.2 |
| BUN (mg/dl) | 2.1 | 2.1 | 2.1 |
| Scr(mg/dl) | 2 | 2 | 2 |
| AST(U/L) | 2.1 | 2.1 | 2.1 |
| ALT(U/L) | 2.1 | 2.1 | 2.1 |
| ALB(g/dL) | 1.7 | 1.7 | 1.7 |
| HBA1c(mmol/L) | 1.7 | 1.7 | 1.7 |
| FIB (mg/L) | 1.3 | 1.3 | 1.2 |
| Sex | 1.8 | 1.8 | 1.7 |
| Age(years) | 1.4 | 1.4 | 1.4 |
| Previous stroke/TIA | 1.1 | 1.1 | 1.1 |
| Hypertension | 1.2 | 1.2 | 1.2 |
| Diabetes | 1.7 | 1.7 | 1.7 |
| Smoking | 1.5 | 1.5 | 1.5 |
| Atrial fibrillation | 1.2 | 1.2 | 1.2 |
| CHD | 1.1 | 1.1 | 1.1 |
| NIHSS score | 1.2 | 1.2 | 1.2 |
| Stroke etiology | 1.1 | 1.1 | 1.1 |

**NA was the excluded variable**

HGB, hemoglobin concentration; HCT, hematocrit; PLT, platelet; TG, triglyceride; TC, total cholesterol; HDL-c, high-density lipoprotein cholesterol; LDL-c, low-density lipoproteins cholesterol; BUN, blood urea nitrogen; Scr, serum creatinine; ALT, alanine aminotransferase; AST, aspartate aminotransferase; ALB, serum albumin; FIB, fibrinogen; BMI, body mass index; CHD, coronary heart disease; TIA, transient ischemia attack. LAA, large artery atherosclerosis; SVO, small vessel occlusion; CE, cardio embolism; NIHSS, national institute of health stroke scale.

**Table S2. Influencing factors of unfavorable outcomes in acute ischemic stroke using univariate regression analysis**

| variable | Characteristics | | OR 95%CI P-value | | |  |
| --- | --- | --- | --- | --- | --- | --- |
| Sex |  | |  | | |  |
| Male | 1166 (61.465%) | | 1.0 | | |  |
| Female | 731 (38.535%) | | 1.651 (1.349, 2.020) <0.001 | | |  |
| Age(years) |  | |  | | |  |
| <60 | 431 (22.720%) | | 1.0 | | |  |
| 60 to <70 | 503 (26.516%) | | 1.133 (0.822, 1.561) 0.446 | | |  |
| 70 to <80 | 668 (35.213%) | | 1.909 (1.429, 2.551) <0.001 | | |  |
| ≥80 | 295 (15.551%) | | 3.998 (2.872, 5.566) <0.001 | | |  |
| HGB（g/dL） | 13.475 ± 2.003 | | 0.821 (0.781, 0.863) <0.001 | | |  |
| HCT (%) | 40.067 ± 5.588 | | 0.933 (0.916, 0.950) <0.001 | | |  |
| PLT (10^9/L) | 223.595 ± 71.264 | | 0.999 (0.998, 1.001) 0.383 | | |  |
| TC (mmol/L) | 4.632 ± 1.123 | | 0.835 (0.762, 0.916) <0.001 | | |  |
| TG (mmol/L) | 1.256 ± 0.639 | | 0.749 (0.631, 0.889) <0.001 | | |  |
| HDL-c(mmol/L) | 1.203 ± 0.350 | | 0.881 (0.662, 1.173) 0.387 | | |  |
| LDL-c(mmol/L) | 2.782 ± 0.959 | | 0.878 (0.790, 0.977) 0.017 | | |  |
| BUN (mg/dl) | 17.611 ± 8.897 | | 1.016 (1.006, 1.027) 0.003 | | |  |
| Scr(mg/dl) | 1.089 ± 1.039 | | 1.017 (0.927, 1.117) 0.717 | | |  |
| AST(U/L) | 26.020 ± 14.191 | | 1.009 (1.003, 1.016) 0.007 | | |  |
| ALT(U/L) | 22.247 ± 15.788 | | 0.993 (0.986, 1.000) 0.037 | | |  |
| ALB(g/dL) | 4.018 ± 0.428 | | 0.272 (0.213, 0.347) <0.001 | | |  |
| HBA1c%) | 6.294 ± 1.114 | | 1.101 (1.010, 1.200) 0.029 | | |  |
| FIB (mg/L) | 333.854 ± 86.410 | | 1.003 (1.002, 1.004) <0.001 | | |  |
| NIHISS score | 5.387 ± 5.718 | | 1.229 (1.202, 1.256) <0.001 | | |  |
| BMI (kg/m^2^) | 23.442 ± 3.152 | | 0.908 (0.879, 0.938) <0.001 | | | |
| Previous stroke/TIA |  | |  | | |  |
| No | 1495 (78.809%) | | Ref | | |  |
| Yes | 402 (21.191%) | | 1.816 (1.440, 2.289) <0.001 | | |  |
| Hypertension |  | |  | | |  |
| No | 692 (36.479%) | | Ref | | |  |
| Yes | 1205 (63.521%) | | 1.345 (1.089, 1.661) 0.006 | | |  |
| Diabetes |  | |  | | |  |
| No | 1287 (67.844%) | | Ref | | |  |
| Yes | 610 (32.156%) | | 1.437 (1.167, 1.771) <0.001 | | |  |
| CHD | |  | |  |  |  |
| No | | 1677 (88.403%) | | Ref |  |  |
| Yes | | 220 (11.597%) | | 1.026 (0.753, 1.398) 0.871 |  |  |
| Atrial fibrillation | |  | |  |  |  |
| No | | 1491 (78.598%) | | Ref |  |  |
| Yes | | 406 (21.402%) | | 2.016 (1.602, 2.537) <0.001 |  |  |
| Smoking | |  | |  |  |  |
| No | | 1149 (60.569%) | | Ref |  |  |
| Yes | | 748 (39.431%) | | 0.612 (0.496, 0.756) <0.001 |  |  |
| Stroke etiology |  | |  | | |  |
| SVO | 365 (19.241%) | | Ref | | |  |
| LAA | 601 (31.682%) | | 1.612 (1.171, 2.218) 0.003 | | |  |
| CE | 492 (25.936%) | | 2.411 (1.748, 3.326) <0.001 | | |  |
| Other determined | 170 (8.962%) | | 3.287 (2.199, 4.914) <0.001 | | |  |
| Undetermined | 269 (14.180%) | | 1.392 (0.948, 2.043) 0.092 | | |  |

Values are mean ± standard deviation or median (quartile) or number (%)

HGB, hemoglobin concentration; HCT, hematocrit; PLT, platelet*;* TG, triglyceride; TC, total cholesterol; HDL-c, high-density lipoprotein cholesterol; LDL-c, low-density lipoproteins cholesterol; BUN, blood urea nitrogen; Scr, serum creatinine*;* ALT, alanine aminotransferase; AST, aspartate aminotransferase; ALB, serum albumin; FIB, fibrinogen; BMI, body mass index; CHD, coronary heart disease; TIA, transient ischemia attack. LAA, large artery atherosclerosis; SVO, small vessel occlusion; CE, cardio embolism; NIHSS, national institute of health stroke score

**Table S3. Relationship between BMI and 3-month unfavorable outcomes in patients with acute ischemic stroke based on marginal structural model analysis**

| Exposure | Model I(OR,95%CI) P | Model II(OR,95%CI) P |
| --- | --- | --- |
| All participants | 0.9834(0.9454,1.0230) 0.4065 | 0.9786 (0.9345, 1.0247) 0.3570 |
| Male | 0.9609 (0.9106,1.003) 0.1460 | 1.0123 (0.9392, 1.0911) 0.7488 |
| Female | 0.9826(0.9339,1.0339) 0.4989 | 0.9690 (0.9153, 1.0259) 0.2797 |

Model I: we adjusted age, sex, smoking, and NIHSS score.

Model II: we adjusted age, sex, LDL-c, HGB, TG, AST, HCT, ALT, BUN, HBA1C, FIB, ALB, previous stroke or TIA, atrial fibrillation, hypertension, smoking, DM, stroke etiology, and NIHSS score.

Note: In the male and female subgroups, Models I and II were not adjusted for the stratification variable sex.

**Figure S1 Distribution of NIHSS score.**

Figure S1. It presented a skewed distribution ranging from 0 to 33 with a median (interquartile) of 3 (1,7).
